# The Effect of an Acidic Food-Simulating Environment on the Shear Bond Strength of Self-Ligating Brackets with Different Base Designs

**DOI:** 10.1155/2014/689536

**Published:** 2014-09-23

**Authors:** Ahmad Sheibaninia, Sepehr Sepasi, Mohammad Ali Saghiri, Setareh Sepasi

**Affiliations:** ^1^Department of Orthodontics, Islamic Azad University, Dental School, Tehran Branch, No. 4, 10th Neyestan Alley, Pasdaran Avenue, P.O. Box 19585-175, Tehran, Iran; ^2^Department of Dental Materials, Islamic Azad University, Dental School, Tehran Branch, No. 4, 10th Neyestan Alley, Pasdaran Avenue, P.O. Box 19585-175, Tehran, Iran; ^3^Department of Biology, University of Massachusetts Amherst, Amherst, MA 01003, USA

## Abstract

*Aim.* This study aims to evaluate the effect of acidic food simulant and (acetic acid 3%) on the shear bond strength (SBS) and adhesive remnant index (ARI) scores of one conventional and three different self-ligating brackets with different base designs. *Materials and Methods.* Freshly extracted first maxillary premolars (*n* = 160) were embedded in resin blocks. A conventional stainless steel bracket, Equilibrium 2, and three types of self-ligating brackets, Speed, In-Ovation R, and Damon 3MX, were bonded to teeth and exposed to distilled water (groups 1, 3, 5, and 7) or acetic acid 3% (groups 2, 4, 6, 8) for 12 weeks. SBS and ARI were calculated and statistical analysis was performed with the analysis of variance (SBS) or *χ* ^2^ test (ARI) to compare values between the different groups. *Results.* Equilibrium 2 and In-Ovation R showed a significantly lower SBS in the acidic environment than in distilled water. Significant differences in ARI scores were found for Equilibrium 2 after immersion in an acidic environment, shifting from 0 in distilled water to 2 in an acidic environment. *Conclusions.* Equilibrium 2 and In-Ovation R brackets showed a significant decrease in SBS after a 12-week immersion in acetic acid 3%, although all groups showed clinically acceptable SBS. Equilibrium 2 showed significant differences in ARI scores when exposed to acetic acid 3%.

## 1. Introduction

One of the greatest concerns during orthodontic treatment is the bonding strength between the bracket and the enamel surface [1–3]. Bonded brackets are routinely used in fixed orthodontic treatments. Thus, achieving adequate bond strength is of great importance in all fixed orthodontic treatments [4]. Through the evolution of adhesive materials, bondable brackets were introduced in orthodontic treatment in 1975 [4]. Reynolds [5] was the first to evaluate the bond strength between brackets and enamel surfaces. Following this, many procedures, such as the proper preparation of enamel [6], increasing the quality of applied adhesives [7], and the use of brackets with retentive base designs [8], have been implemented to improve the shear bond strength (SBS) of brackets. If adequate bond strength is not achieved, debonding will occur and the bracket will need to be replaced, a process which is time consuming and can incur an extra cost for the patient.

Most of the previous studies evaluating the SBS of brackets used conventional stainless steel brackets [1–3, 9], and only a few published studies have evaluated the SBS of self-ligating brackets [6, 7, 10, 11]. Further, both the oral environment and the brackets are exposed to acidic food and drinks over the duration of orthodontic treatment. However, nearly all of the previous studies evaluating SBS used distilled water as the storage solution for the brackets being tested and articles evaluating the effect of acidic simulants on the SBS of conventional brackets are scarce [12, 13]. There is, to date, no study evaluating the effect of an acidic environment on the SBS of self-ligating brackets.

Therefore, the aim of the present study was to evaluate,* in vitro*, the effect of acidic food simulant on the SBS and adhesive remnant index (ARI) score of three different self-ligating brackets and one conventional bracket with different base designs. The null hypothesis of this study was that there are no significant differences in SBS values and debonding location between the various groups when exposed to an acidic food simulant environment.

## 2. Materials and Methods

One hundred and sixty freshly extracted first maxillary premolars were collected from orthodontic patients with an indication of premolar extraction and were stored in thymol for 48 h. All the teeth tested in this study had no signs of caries, cracks, or hypocalcification. The teeth were embedded in acrylic resin in such a way that their facial surface would be exposed. A mounting jig was used to parallel the facial surface of each tooth to the force that would be applied later. Acetic acid 3% was used as an acidic food simulant as recommended by the US food and drug administration [14]. One conventional bracket, Equilibrium 2 (Dentaurum GmbH & Co., Ispringen, Germany), and three different self-ligating brackets, Speed (Strite Industries Ltd., Cambridge, ON, Canada), In-Ovation R (DENTSPLY GAC International, Islandia, NY, USA), and Damon 3MX (Ormco Corporation, Orange Co., CA, USA), were tested. A set of all four bonded brackets was divided to eight groups of 20; groups 1, 3, 5, and 7 were stored in distilled water for 12 weeks and other groups 2, 4, 6, and 8 in acetic acid 3% for 12 weeks before testing the SBS. Teeth were bonded and tested according to the guidelines described by Fox et al. [15]. In brief, facial surfaces of teeth were cleaned by rubber cup and pumice with a low speed hand-piece for 10 s. The teeth were then cleaned with water and were air-dried to remove the pumice remnants off the tooth surface.

Teeth were etched for 30 s with phosphoric acid gel (3M Unitek, Monrovia, CA, USA), water was sprayed for 20 s, and dried with an oil-free air spray. A layer of 3M Unitek bonding primer was applied to the etched surfaces with a brush. An adhesive (3M Unitek Transbond XT) was applied to the base of the brackets and brackets were placed on the prepared enamel surfaces in the center of the facial surfaces along the long axes of the teeth. A scaler was used to exert force on the brackets and excess adhesive was cleaned from the margin of the base of the brackets. For adhesive polymerization, brackets were lighted by a Starlight Pro LED light curing unit (Mectron, Carasco, AG, Italy) for 10 s from the mesial side and 10 s from the distal side.

After immersion in either acetic acid 3% or distilled water for 12 weeks, shear force was exerted by a Zwick/Roell Z20 universal testing machine (Zwick GmbH and Co, Germany) at the speed of 1 mm/min at the bracket base-tooth interface, as in previous studies [10, 16]. The maximum load at which the bracket was debonded was recorded in Newtons and the data was converted to MPa by dividing it by the surface area of the base of each bracket. A light stereomicroscope (Carl Zeiss, Oberkochen, Germany) with 10x magnification was used to examine the enamel surfaces for residual adhesive after debonding. The values were given according to the ARI, as described by Årtun and Bergland [17], as follows: 0, no adhesive left on the tooth; 1, less than 50% of adhesive left on tooth; 2, more than 50% of adhesive left on the tooth; and 3, all adhesives left on the tooth.

Normality of the data was evaluated by the Kolmogorov-Smirnov test. Analysis of variance (Anova) and* post hoc* Tukey test were used to evaluate the differences in SBS values between the study groups. The *χ* ^2^ test was used to evaluate any significant differences with regard to the ARI results.

## 3. Results

Descriptive statistics for the SBS of the various groups of brackets are shown in Table 1. Normality of the data was calculated by the Kolmogorov-Smirnov test; Anova showed a significant difference among the various groups. The Equilibrium 2 and Speed brackets demonstrated the highest and lowest SBS values, respectively, in distilled water. The* post hoc* test showed a significant decrease in SBS values for the Equilibrium 2 and In-Ovation R brackets in the acidic environment (groups 2 and 6) compared to distilled water (groups 1 and 5) (Equilibrium 2, *P* < 0.05; In-Ovation R, *P* < 0.05). Equilibrium 2 brackets in distilled water showed a significantly higher SBS value compared to all other groups (*P* < 0.05). The Speed and Equilibrium 2 brackets demonstrated the highest and lowest SBS values, respectively, in the acidic environment. Among all 160 specimens, only one sample (In-Ovation R in the acidic group) had an SBS value less than 6 Mpa.

The ARI scores are shown in Table 2. The *χ* ^2^ test showed a higher frequency of ARI score 1 for the Speed, Damon 3MX, and In-Ovation R brackets in both the acidic and distilled water environments without any significant difference between them. Equilibrium 2 showed a significant difference in ARI score when exposed to acetic acid 3%, with a higher frequency of ARI score 0 in distilled water and a higher frequency of ARI score 2 in the acidic environment.

## 4. Discussion

**Figure 1:**
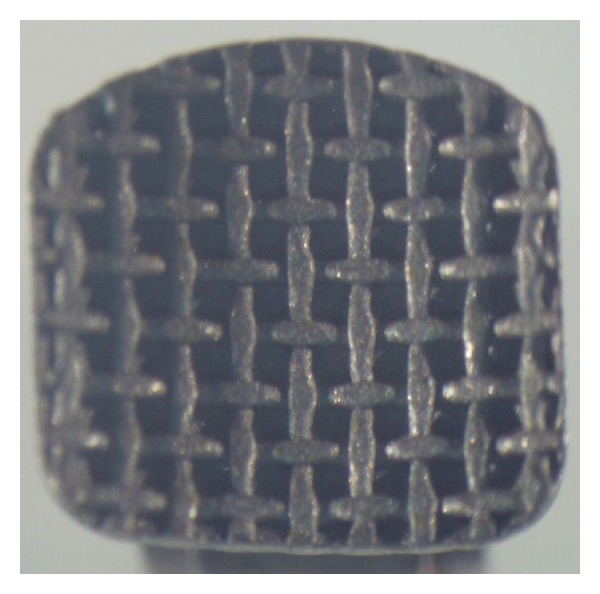
Speed bracket, single layer (100x magnification).

**Figure 2:**
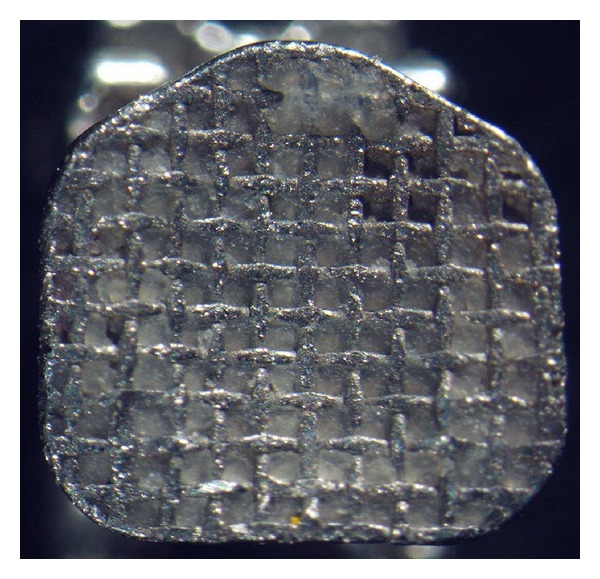
Damon 3MX bracket, single layer.

**Figure 3:**
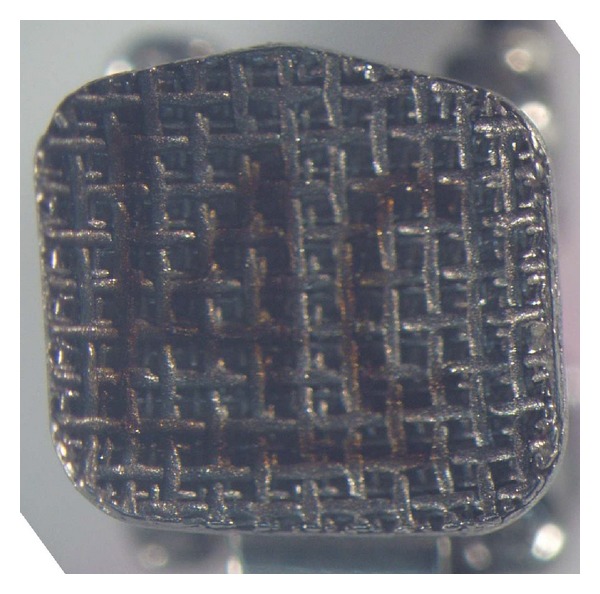
In-Ovation R bracket, double layer.

As can be observed from the obtained results, the null hypothesis of the study was rejected. The Equilibrium 2 and In-Ovation R brackets showed significantly lower SBS values when exposed to an acidic environment. Further, the ARI score showed a significant difference for the Equilibrium 2 brackets after immersion in acidic food simulant. These results are probably due to the differences in base design; there is a strong relationship between bracket base design and SBS value [8]. In the current study, all three types of self-ligating brackets had a foil mesh pattern base design: the Speed bracket has a 60-gauge single-layer foil mesh (Figure 1), Damon 3MX has a 100-gauge single-layer OptiMesh (Figure 2), and In-Ovation R has a double-layer foil mesh comprising an 80-gauge foil mesh brazed into a 150-gauge layer (Figure 3). It has been indicated that the adhesive composite cannot fully penetrate into the double-layer foil mesh design to the same extent that it can penetrate a single-layer foil mesh [8]. This incomplete adhesive composite penetrance allows the acid to penetrate the bracket base-adhesive interface, thus lowering the SBS value in the In-Ovation R brackets.

**Figure 4:**
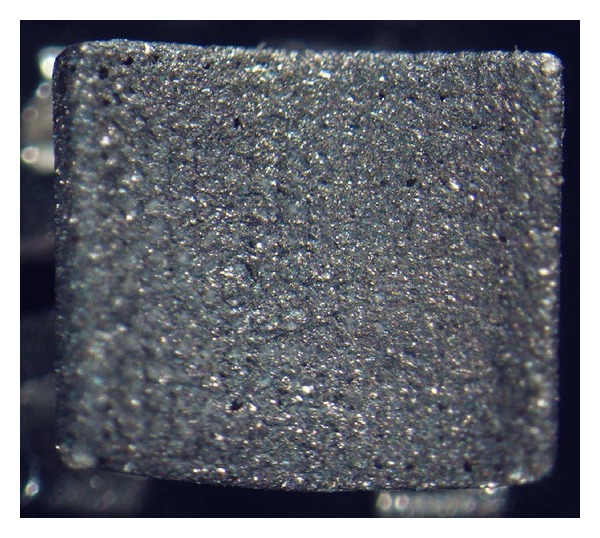
Equilibrium 2 bracket, laser-base.

The Equilibrium 2 conventional bracket is a one-piece metal injection molded bracket with laser-created retentive grooves at the base. This uniform, highly dense retentive area is responsible for the highest mean SBS value observed for the Equilibrium 2 brackets after a 12-week immersion in distilled water. However, the significant reduction observed in SBS value after acid immersion may be due to its rectangular shape (Figure 4), which does not fully adapt to the 3D shape and curved contour of the facial surface of upper first premolar teeth, particularly at the margins of the bracket base, thus making it a vulnerable point for acid penetrance.

Previous studies regarding the acidic effects of food simulants on SBS are scarce, and those available did not find any effect of acid immersion on SBS value. Vicente et al. [13] evaluated the SBS value of one conventional bracket (Victory 3M) after thermocycling and immersion in acetic acid 3% using either HEMA-free or HEMA-containing self-etching primer and demonstrated no significant difference in SBS value after acid immersion. The Victory 3M bracket is an 80-gauge single-layer foil mesh bracket, which is probably why the SBS value was unaffected and showed a similar behavior to the Speed and Damon 3MX brackets in our study. Hobson et al. [12] evaluated the SBS value of one conventional bracket in different types of food simulant environments and observed no significant difference in the SBS value of these brackets when exposed to lactic acid (pH 4) for 3 months.

All of the brackets in this study showed a bond strength above the least required bond strength for orthodontic applications (6 to 8 MPa) [5]. Further, Newman et al. [18] stated that the SBS value should be under 21 MPa to avoid enamel tear-out. However, in this study, the Equilibrium 2 bracket had a mean SBS value of 27.11 ± 1.16 MPa, which is above the maximum recommended limit. Nevertheless, upon microscopic evaluation, no cracks were observed on the enamel surface following the debonding of the Equilibrium 2 brackets.

In this study, we demonstrated that there is a high frequency of ARI score 1 in both water and acid environments for all self-ligating brackets. Equilibrium 2 showed a significant difference in ARI scores between distilled water and acid environments. Further, immersion in acid tended to shift the bond failure location from the adhesive-enamel interface toward the adhesive-bracket interface. Previous studies evaluating the SBS of self-ligating brackets after 24 h immersion in water stated a higher frequency of ARI score 3 [6, 7] and ARI scores 1 and 2 [10]. An ARI score of 0 indicates that bond failure occurs between the composite and enamel, which is less time consuming regarding the removal of adhesive from the tooth surface following the debonding procedure, although it carries a greater risk of enamel tear-out. An ARI score of 3 indicates that all the adhesives remained on the tooth surface, with less risk of enamel tear-out but more time required to remove the adhesive bulk [11].

## 5. Conclusions

Equilibrium 2 and In-Ovation R brackets showed a significant decrease in SBS values when exposed to acidic food simulant. All brackets showed the required SBS values for clinical applications in both water and acidic environments. The Equilibrium 2 brackets showed a significant difference in ARI scores between distilled water and acidic environments.

## Figures and Tables

**Table 1:** Descriptive statistics of the four groups tested (MPa).

Group	Storage condition	Number of observations	Mean	SD^a^	Minimum	Median	Maximum	Tukey grouping^b^
1 (Equilibrium 2)	Water	20	27.11	1.16	10.86	25.98	49.12	A
2 (Equilibrium 2)	Acetic acid 3%	20	11.31	3.93	6.30	10.54	18.80	BE
3 (Speed)	Water	20	15.99	4.06	9.64	16.72	24.16	CB
4 (Speed)	Acetic acid 3%	20	14.30	4.32	6.04	14.81	21.29	CB
5 (In-Ovation R)	Water	20	18.56	5.87	9.92	19.25	32.09	DC
6 (In-Ovation R)	Acetic acid 3%	20	11.37	3.52	7.09	12.48	18.56	BE
7 (Damon 3MX)	Water	20	16.22	5.87	7.83	14.47	32.70	CE
8 (Damon 3MX)	Acetic acid 3%	20	14.23	3.23	7.79	13.14	20.51	CB

^a^SD: standard deviation.

^b^Tukey grouping: means that do not share a letter are significantly different.

**Table 2:** Frequency of distribution of adhesive remnant.

Group	Condition	ARI = 0, number (%)	ARI = 1, number (%)	ARI = 2, number (%)	ARI = 3, number (%)
1 (Equilibrium 2)	Water	11 (55)	6 (30)	3 (15)	0 (0)
2 (Equilibrium 2)	Acetic acid 3%	4 (20)	4 (20)	7 (35)	5 (25)
3 (Speed)	Water	2 (10)	15 (75)	3 (15)	0 (0)
4 (Speed)	Acetic acid 3%	0 (0)	14 (70)	4 (20)	2 (10)
5 (In-Ovation R)	Water	5 (25)	13 (65)	2 (10)	0 (0)
6 (In-Ovation R)	Acetic acid 3%	2 (10)	12 (60)	4 (20)	2 (10)
7 (Damon 3MX)	Water	0 (0)	14 (70)	3 (15)	3 (15)
8 (Damon 3MX)	Acetic acid 3%	1 (5)	13 (65)	4 (20)	2 (10)
